# 

**Published:** 1911-12

**Authors:** 


DECEMBER, 1911.

Vol. XXIX. No. 114.

### THE BRISTOL MEDICO-CHIRURGICAL JOURNAL

PUBLISHED UNDER THE AUSPICES OF

*THE BRISTOL MEDICO-CHIRURGICAL SOCIETY.*

LIBRARY
JAN. 5 1912
SURGEON-GENERAL'S OFFICE

*Editor:* R. SHINGLETON SMITH, M.D., B.Sc.

WITH WHOM ARE ASSOCIATED

J. MICHELL CLARKE, M.A., M.D.,

J. LACY FIRTH, M.S., M.D., JAMES SWAIN, M.S., M.D.

P. WATSON WILLIAMS, M.D., *Assistant Editor.*

*Editorial Secretary:* J. A. NIXON, B.A., M.B.

BRISTOL: J. W. ARROWSMITH LTD.

LONDON: J. & A. CHURCHILL, 7 GREAT MARLBOROUGH STREET.

*Price One Shilling and Sixpence.*

